# Peroxiredoxins in the Developmental Origins of Health and Disease: Insights from Maternal Under and Overnutrition – A Critical-Interpretative Scoping Review

**DOI:** 10.1007/s12013-026-02061-9

**Published:** 2026-04-06

**Authors:** Matheus Naia Fioretto, Luísa Correia Gomes, Flávia Alessandra Maciel, Ana Luiza Silva de Abreu, Ana Luiza Romano Gabriel, Sérgio Luis Felisbino, Luis Antonio Justulin

**Affiliations:** Department of Structural and Functional Biology, Institute of Biosciences, São Paulo State University, Botucatu, SP Brazil

**Keywords:** Biological Mechanisms, Biomarkers, DOHaD, Maternal Malnutrition, Peroxiredoxins

## Abstract

Epidemiological and experimental evidence indicate that disturbances during pregnancy or lactation can induce metabolic programming in offspring, increasing lifelong disease risk. Maternal malnutrition, whether by excess or deficiency, is linked to metabolic disorders such as type 2 diabetes, obesity, and cardiovascular diseases. Current research aims to identify biomarkers of the developmental origins of health and disease (DOHaD). Among these, peroxiredoxins (PRDX1–6) have gained attention for their mitochondrial, cytoplasmic, and endoplasmic reticulum roles in antioxidant defense, inflammation, and energy metabolism. This scoping review aimed to understand what is known about the effects of maternal malnutrition on the PRDXs in different organs in the offspring, to understand whether these biomolecules have a potential role in the DOHaD context. This review followed PRISMA-ScR guidelines and systematically searched PubMed studies linking maternal nutritional undernutrition or overnutrition to PRDX expression in offspring. Only studies directly assessing PRDXs after maternal dietary interventions were included; reviews, preprints, and studies without maternal influence were excluded. Therefore, we identified 426 manuscripts, of which 346 were removed for being outside the scope of the review, 41 for being duplicates, 1 for not reporting the effect on PRDXs, 21 for not being related to maternal diet, 2 were review articles, and 1 was used as a model of maternal stress. Therefore, a total of 14 manuscripts were selected, all of an experimental nature. Maternal malnutrition affects different PRDX isoforms in several models and organs, outlining possible disorders associated with cellular function and stress response. Therefore, maternal undernutrition affects cardiac, cerebral, hepatic, ovarian, pancreatic, and adrenal PRDXs, while maternal overnutrition affects PRDXs in the salivary glands, intestine, and ovaries, establishing possible metabolic and reproductive risks through biological mechanisms that still need to be thoroughly investigated. We highlighted numerous gaps in the literature and provided a solid foundation for future experimental and translational studies that will establish whether PRDXs are biomarkers of the developmental origins of diseases and maternal malnutrition.

**Supplementary Information:**

The online version contains supplementary material available at https://doi.org/10.1007/s12013-026-02061-9.

## Introduction

The Developmental Origins of Health and Disease (DOHaD) concept proposes that adverse conditions during critical developmental windows, such as the intrauterine and early postnatal environment, can cause permanent structural and functional alterations in specific organs, increasing susceptibility to chronic noncommunicable diseases (NCDs) later in life [1–3] - a concept established by metabolic-epigenetic programming. One of these conditions may be dietary, and according to the World Health Organization, malnutrition - including undernutrition, micronutrient deficiencies, overweight, obesity, and related NCDs - remains a critical global health challenge. In 2022, 2.5 billion adults were overweight, including 890 million with obesity, while 390 million were underweight - contexts that also affect children and early life [4]. Improving maternal and adolescent nutrition requires multisectoral strategies - ensuring access to nutritious and affordable foods; reducing exposure to ultra-processed products; strengthening nutrition and social protection services; and implementing gender-transformative policies that combat discrimination and empower women to exercise their right to adequate nutrition [5].

Maternal malnutrition, characterized by the depletion of macro- and/or micronutrients, remains a major global health concern affecting women during pregnancy and lactation. Epidemiological evidence indicates that maternal malnutrition around conception is associated with cognitive impairments and a higher risk of psychiatric disorders in offspring, including depression and schizophrenia [6]. Likewise, choline deficiency during pregnancy has been linked to abnormal cognitive development in children [7]. In humans, maternal protein restriction (MPR) has been shown to affect the prefrontal cortex of offspring, increasing the risk of depression, dementia, and memory disorders [8, 9]. Experimentally, MPR—one of the most widely used models to simulate food insecurity and nutritional deprivation—has been shown to impair reproductive [10], renal [11, 12], cardiorespiratory [13, 14], hepatic [15–17], gastrointestinal [18], muscular [19], and endocrine [20] systems at various stages of development, ultimately increasing susceptibility to metabolic and chronic diseases later in life.

Conversely, maternal overnutrition often entails excessive intake of specific macronutrients (e.g., lipids or carbohydrates), which has been linked to an increased risk of metabolic disorders and chronic diseases [21, 22]. Cohort studies indicate that a higher pre-gestational body mass index elevates the risk of hypertension in offspring [22]. Moreover, maternal overnutrition impairs insulin sensitivity [23] and predisposes offspring to overweight and obesity [24]. In rats, maternal overnutrition before and during gestation induces chromosomal instability in male offspring and promotes oxidative stress and hypercholesterolemia in females [25]. Experimental models further show that maternal cafeteria diets reduce sperm quality in offspring [26]. Additionally, maternal high-fat diets during pregnancy and lactation increase depressive-like behaviors, impair cognitive development, and trigger metabolic syndrome symptoms [27], while also disrupting hepatic cholesterol metabolism [28].

In this context, it is essential to elucidate how maternal undernutrition or overnutrition programs offspring at the molecular level, particularly with respect to the expression and functional regulation of PRDX isoforms. Alterations in PRDX dynamics may differentially affect mitochondrial, cytoplasmic, and endoplasmic reticulum, influencing redox signaling, antioxidant defenses, and inflammatory pathways. Such disturbances are unlikely to remain confined to isolated cellular events; rather, they may propagate to tissue remodeling and ultimately contribute to systemic dysfunction, reinforcing the potential relevance of PRDXs as molecular mediators of developmental programming.

Therefore, this review is based on 3 central questions:

1. Given the diverse roles of PRDXs across cellular compartments, molecular pathways, and physiological and pathological contexts, this review seeks to expand current understanding by synthesizing available evidence and outlining future research directions.
2. Considering the importance of the maternal environment and the potential outcomes of maternal malnutrition on offspring, this review also aimed to articulate how such conditions affect the offspring, from the perspective of a broad and growing area – DOHaD.
3. As a third pillar of this review, we aim to articulate the two aforementioned contexts to understand the current state of knowledge regarding maternal malnutrition and PRDXs in specific organelles and organs of offspring.

Therefore, the objective of this critical and interpretative scoping review was to examine the current body of knowledge on how maternal malnutrition or overnutrition influences the expression of distinct PRDX isoforms across several cells, tissues, and organs in offspring, to elucidate their role in fetal metabolic programming and identify potential convergent adverse effects related to PRDX functions.

## Review Methods

### Generak Protocol

The protocol for this review was based on PRISMAScR (Preferred Reporting Items for Systematic Reviews and Meta-Analyses Extension for Scoping Reviews) guidelines (https://www.prisma-statement.org/scoping / *Supplementary Table **1*) [29]. 

### Search Strategy

The literature search was conducted solely on the PubMed platform (https://pubmed.ncbi.nlm.nih.gov/), considering only articles published in English. PubMed was selected as the primary database due to its strong curation of biomedical, experimental, and translational research, which aligns with the scope of this review. During the search, we used the following descriptors: *Maternal nutrition and peroxiredoxin; Maternal malnutrition and peroxiredoxin; Maternal diet and peroxiredoxin; Maternal protein restriction and peroxiredoxin; Maternal undernutrition and peroxiredoxin; Maternal low protein diet and peroxiredoxin; Maternal overnutrition and peroxiredoxin; Maternal high fat diet and peroxiredoxin; PRDX and nutrition; PRDX and diet; Peroxiredoxin and nutrition; Peroxiredoxin and diet*. The Boolean operator used during study selection was just “AND” to find manuscripts that associated the two correlations. The most recent search date was October 19, 2025. Studies were selected irrespective of publication year due to the limited number of investigations directly examining the effects of maternal malnutrition—whether undernutrition or overnutrition—on PRDX expression in offspring. The search strategy and all applied filters were systematically documented and exported to Excel for organization and transparency. Duplicate records were subsequently removed, after which a structured inclusion and exclusion process was conducted based on predefined eligibility criteria.

### Eligibility and Study Selection Criteria

Based on the predefined sources of evidence, we included original research articles that explicitly investigated the (1) direct impact of maternal malnutrition—either overnutrition or undernutrition—during pregnancy and/or lactation on (2) offspring tissues, and that (3) reported alterations in at least one PRDX isoform. Eligible studies were required to demonstrate a clear experimental design linking maternal dietary intervention to measurable changes in PRDX expression or activity in specific offspring organs.

**Fig. 1:**
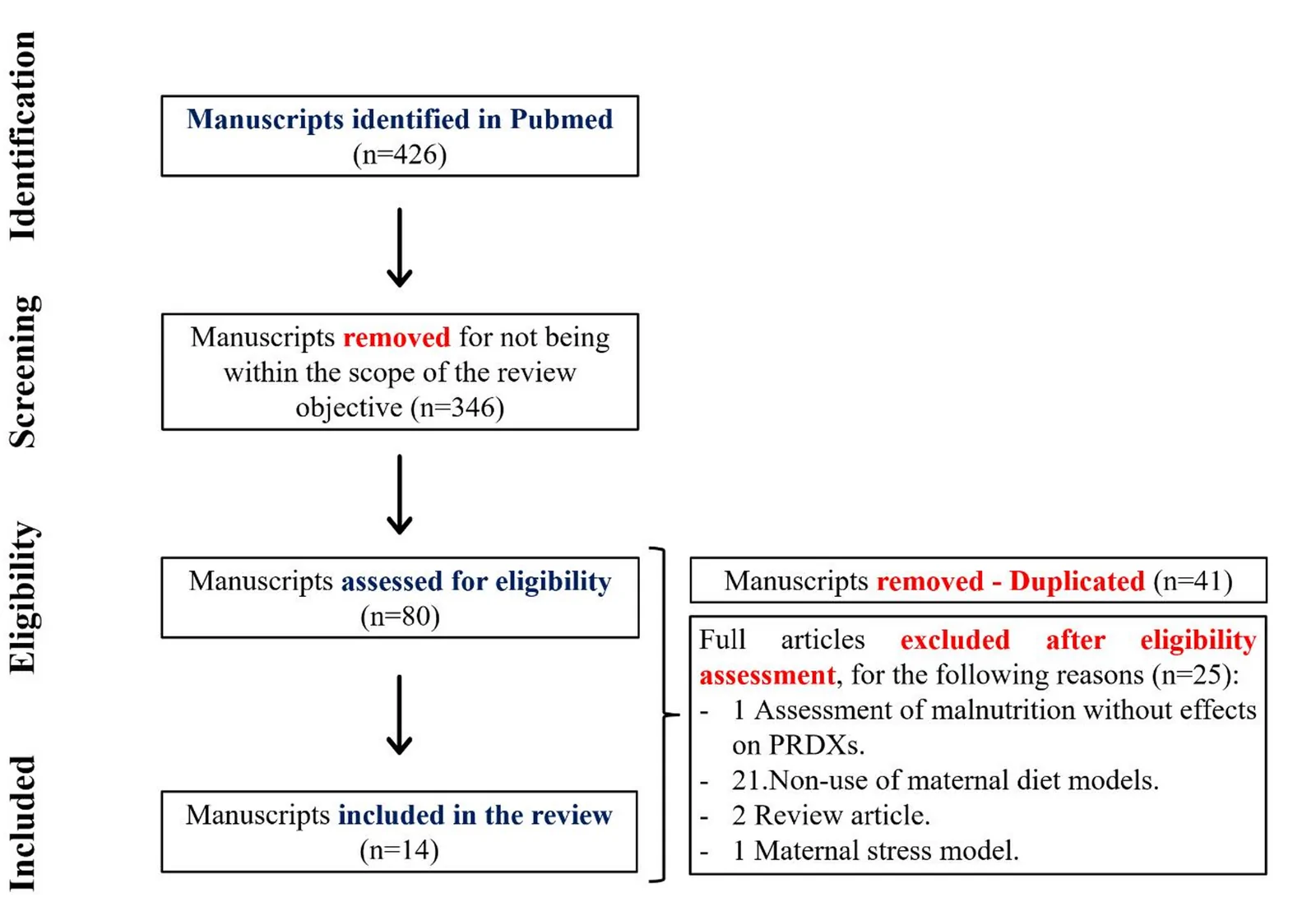
Flow diagram describing the information criteria adopted during the systematic scoping review on maternal overnutrition or undernutrition and Peroxiredoxins (PRDXs)

Exclusion criteria comprised studies that: (1) did not establish a direct association between maternal nutritional status and PRDX modulation in offspring; (2) reported alterations in PRDXs without a maternal dietary component; or (3) lacked a defined nutritional challenge during gestation and/or lactation. Additionally, meta-analyses, preprints, retracted publications, narrative or systematic reviews, scoping reviews, scientific integrity reviews, non-peer-reviewed reports, and comparative articles were excluded to ensure the inclusion of original, peer-reviewed experimental evidence only (Fig. 1).

### Data Extraction and Analysis

During data extraction and collection, two authors (MNF and LCG) performed the screening, spreadsheet organization, and manuscript information and descriptions (main outcomes associated with PRDXs, exposure, period, study design, and authors). Subsequently, the inclusion or exclusion of each article was discussed and analyzed critically and collectively among all authors, establishing the conclusion criteria with the agreement of all authors. Study quality assessment was conducted concurrently throughout this process, but without determining the risk of bias due to the scarcity of studies within the established topic. Although a formal risk-of-bias tool was not applied, all authors reached unanimous agreement during the selection and inclusion process after conducting a rigorous qualitative appraisal of each study. This evaluation considered the methodological structure of the manuscripts, the scientific rigor and transparency of the experimental design, the credibility and indexing of the journals in which they were published, and the overall consistency and reliability of the reported findings. Based on this collective and critical assessment, the included studies were deemed methodologically sound and appropriate for discussion within the scope of this review.

### Functional and Subcellular Characterization of the Peroxiredoxin Isoforms

To associate our observations with future insights, we performed in silico analyses of the 6 PRDX isoforms (PRDX1-6) on the Enrichr platform (https://maayanlab.cloud/Enrichr/) [30–32], using the Reactome Pathways 2024 database to check the 10 most enriched biological pathways, COMPARTMENTS Experimental 2025 to understand the enriched cellular regions, and CellMarker 2024 to generally assess cells that express these PRDXs - considering the p-value significance. For the cellular and cell enrichment, we used *Appyter software* for graph construction (https://appyters.maayanlab.cloud/#/), and considered the -Log10(p-value)/Odds Ratio and UMAP in the graphics, respectively. These results were used as the basis for the insights provided.

## Results

During the search and filtering criteria, we found 426 articles. 346 articles were excluded because they were out of our central scope. Subsequently, we analyzed 80 manuscripts, and removed 41 (duplicated) and 25 for the following reasons: 1 Assessment of malnutrition without effects on PRDXs, 21 Non-use of maternal diet models, 2 Review articles, and 1 Maternal stress model. Therefore, we included 14 articles, 11 associated with maternal undernutrition and PRDXs [14, 17, 20, 33–40] (Table 1) and 3 associated with maternal overnutrition and PRDXs [41–43] (Table 2). The main results, exposition, macromolecules and percentage of diet, period, model, and organ are listed in Tables 1 and 2. 

Based on the selected evidence, we observed that all eligible studies were experimental in nature, underscoring a significant gap in translational and clinical investigations addressing this topic. Collectively, the included studies employed diverse maternal nutritional models—undernutrition or overnutrition—applied during gestation, lactation, or both periods, and evaluated outcomes across multiple stages of developmental biology, including fetal life, post-weaning, adulthood, and aging – with sex-specific effects. In models of maternal undernutrition, reported effects involved the brain, heart, liver, pancreas, ovary, and adrenal glands, with consistent modulation of PRDX isoforms 1–5. Notably, PRDX6 remains largely unexplored in this context, highlighting an important gap in the literature. Conversely, studies investigating maternal overnutrition primarily reported alterations in the intestine, ovary, and salivary glands of the offspring, with predominant involvement of PRDX3, PRDX4, and PRDX6, while evidence regarding PRDX1, PRDX2, and PRDX5 remains limited. Taken together, these findings reveal organ-specific and isoform-specific patterns of PRDX modulation depending on the type of maternal nutritional insult, while also exposing clear gaps that warrant further mechanistic and translational investigation.

**Fig. 2:**
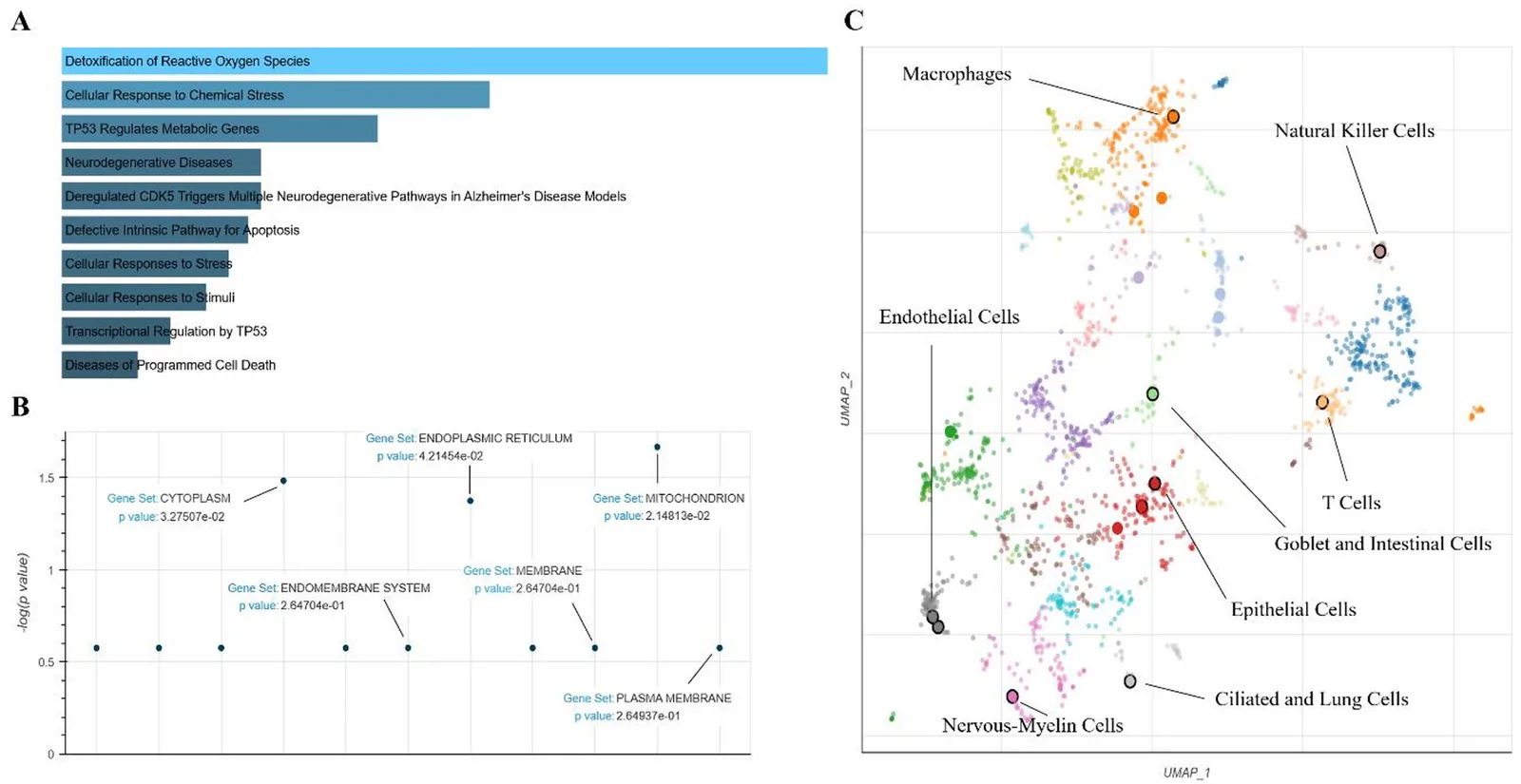
Functional and subcellular characterization of the different Peroxiredoxin isoforms (PRDX 1–6). Functional enrichment analysis revealed significant modulation of pathways associated with oxidative stress response, metabolism, and neurodegenerative processes (**A**). Subcellular localization showed predominant enrichment in mitochondria, cytoplasm, and endoplasmic reticulum (**B**). Cellular mapping indicated higher expression in macrophages, epithelial cells, and intestinal goblet cells, highlighting cell-type-specific oxidative and metabolic adaptations (**C**). Analyses were performed on the Enrichr platform, considering significance through the p-value, and graphs were constructed considering -log(p-value) (**B**) and Uniform Manifold Approximation and Projection (UMAP) (**C**)

We performed in silico analyses for 6 PRDX isoforms to understand their potential biological roles, as well as organellar and cellular aspects. We observed enrichment of biological processes such as: Detoxification of Reactive Oxygen Species, Cellular Response to Chemical Stress, TP53 Regulation, Metabolic Genes, Neurodegenerative Diseases, Deregulated CDK5 Triggers Multiple Neurodegenerative Pathways in Alzheimer’s Disease Models, Defective Intrinsic Pathway for Apoptosis, Cellular Responses to Stress, Cellular Responses to Stimuli, Transcriptional Regulation by TP53, and Diseases of Programmed Cell Death (Fig. 2A). In cellular processes, we observed the Cytoplasm, Endoplasmic Reticulum, Mitochondrion, Endomembrane System, Membrane, and Plasma Membrane (Fig. 2B). In addition, we observed general cell enrichment by Macrophages, Natural Killer Cells, Endothelial Cells, T Cells, Goblet and Intestinal Cells, Epithelial Cells, Ciliated and Lung Cells, and Nervous-Myelin Cells (Fig. 2C). All these results were used to guide us in understanding the observed effects and generated future perspectives about potential PRDX deregulations caused by maternal malnutrition.

## Maternal Undernutrition

Maternal malnutrition encompasses a broad spectrum of nutritional imbalances, including micronutrient deficiencies, inadequate intake of specific macronutrients (e.g., proteins and polyunsaturated fatty acids), and severe caloric restriction. Within the DOHaD framework, elucidating how these early-life nutritional insults shape offspring physiology and disease susceptibility has critical importance. Accordingly, the identification of reliable molecular biomarkers that link maternal nutritional status to long-term health outcomes has become an expanding area of investigation, driven by advances in molecular and omics technologies. In this context, we highlight the modulation of distinct PRDX isoforms as potential biomarkers, summarizing below the evidence regarding how maternal malnutrition influences their expression and function across different developmental stages and tissues.

Male offspring rats exposed to MPR during gestation and lactation exhibited reduced hepatic reticular fibers, mast cell density, glutathione, MDA levels, and PRDX1 expression. In contrast, females showed decreased reticular fibers and downregulated PRDX4 expression. In silico analyses indicated that the predominant alterations in males involved oxidative stress, glycogen metabolism, and inflammatory pathways, whereas females displayed dysregulation of antioxidant mechanisms within the extracellular matrix [17]. In the same model, Fioretto et al. (2025) demonstrated that male offspring rats showed a reduction in the thickness of the fasciculate and reticular zones of the adrenal gland, as well as in the medulla. Furthermore, there was a decrease in PRDX3 and p62 protein expression, increased GSH and CAT levels, and elevated aldosterone synthase (*Cyp11b2*) expression early in life. At postnatal day (PDN) 540, there was a structural adrenal disorder, in addition to reduced PRDX3, PRDX4, and ABCA1 expression, along with increased BAX levels. Additionally, Cyp11b2 expression was elevated, whereas *Cyp11b1* expression and aldosterone levels were decreased, with increased corticosterone levels [20].

In a model of maternal protein or energy restriction, newborn goats exhibited decreased SOD activity in the liver, thymus, and spleen, and reduced CAT activity in the spleen. At 6 weeks of age, SOD activity in the thymus and spleen increased, whereas CAT activity in the thymus remained lower. Thymic *Sod1* mRNA expression was also reduced. At 22 weeks, *Prdx2* and *Cat* mRNA expression in the liver was decreased. Overall, maternal malnutrition - whether due to protein or energy restriction - compromised the antioxidant capacity of neonatal offspring. Although most antioxidant enzyme activities and gene expressions in plasma and immune tissues recovered after 6 weeks (males) and 22 weeks (females) of nutritional rehabilitation, the findings indicate persistent programming effects on the offspring [33].

In brain tissue, MPR reduced the expression of α-synuclein and PRDX2; however, these alterations were reversed following taurine supplementation. In contrast, lactate dehydrogenase B chain, SUMO-activating enzyme subunit 1, and creatine kinase B-type were elevated in the restricted offspring group. Pathway Analysis of functional associations within these proteins revealed strong links to neurological diseases and to nervous system development and function, including Parkinson’s signaling and glutathione-related redox reactions. The authors concluded that taurine is essential for normal growth and development of the nervous system [34], and can reverse the negative effects of MPR.

Maternal undernutrition significantly reduced birth weight in the offspring, decreasing both primordial, secondary, and antral follicle numbers. In chow-fed offspring, ovarian *Gdf9* mRNA levels were significantly lower, in addition to increased *Fshr* mRNA levels. In contrast, *Er-β *mRNA expression was significantly reduced. Maternal undernutrition also increased hyperoxidized ovarian *Prdx3* levels and decreased ovarian *Prdx3* mRNA expression [35].

MPR reduced fetal and neonatal body weight and insulin levels, accompanied by decreased antioxidant enzyme activity. Pancreatic islets showed persistently low SOD and minimal CAT activity, while hepatic SOD and GPX were also reduced. PRDX activity was absent in fetal tissues and decreased postnatally, with PRDX1 downregulated and PRDX2 slightly increased early in life. Reduced PRDX5 activity in the liver and unbalanced islet SOD activity without CAT/GPX compensation indicates oxidative stress, linked to increased c-Myc, unchanged PDX-1, and reduced insulin mRNA expression [36]. MPR also caused significantly shorter telomeres in pancreatic islets, leading to increased expression of PRDX1, PRDX3, and heme oxygenase 1 (HO-1) in the offspring. In contrast, MnSOD expression was reduced, indicating a partial impairment of mitochondrial antioxidant defenses. Moreover, cellular senescence markers, p21 and p16, were increased in, suggesting premature cellular aging in pancreatic islets. These findings may contribute to β-cell dysfunction and increased susceptibility to type 2 diabetes later in life [37].

In the same model, intrauterine growth restriction followed by accelerated postnatal growth increased oxidative and nitrosative stress. Postnatal CoQ10 supplementation corrected these antioxidant imbalances in the heart. In recovered offspring, MnSOD, CuZnSOD, and CAT levels increased at 12 months, though CoQ10 delayed their elevation. HO-1 levels were elevated in offspring of nutrient-restricted dams and remained unaffected by CoQ10. Conversely, PRDX3 was reduced in recovered animals, an effect prevented by CoQ10, while GPX1 showed the opposite pattern - elevated in recovered offspring and further increased by CoQ10 [38]. In consonance, MPR led to cardiac tissue increased protein nitrotyrosination and single-stranded DNA breaks. At postnatal day 3, DNA repair enzymes 8-oxoguanine-DNA-glycosylase-1, nei-endonuclease-VIII-like, X-ray-repair-complementing-defective-repair-1, and Nthl endonuclease III-like-1 were upregulated, with persistent and more pronounced differences for some enzymes at day 22. Xanthine oxidase and NADPH oxidase were elevated, while antioxidant enzymes PRDX3 and CuZn-superoxide-dismutase were reduced. These findings indicate that low birth weight followed by rapid postnatal growth promotes cardiac oxidative and nitrosative stress and DNA damage, which may contribute to increased cardiovascular risk [39].

Sobrinho Lemos & Naia Fioretto et al. (2025) recently demonstrated that MPR impacts serum levels of IGF1 and testosterone, leading to a structural decrease in components of the cardiac extracellular matrix and microvasculature. These impacts caused cardiac electrophysiological disturbances (bradycardia), in addition to affecting proteins associated with the antioxidant system (low levels of PRDX4 and high levels of GSTpi) [14].

**Fig. 3:**
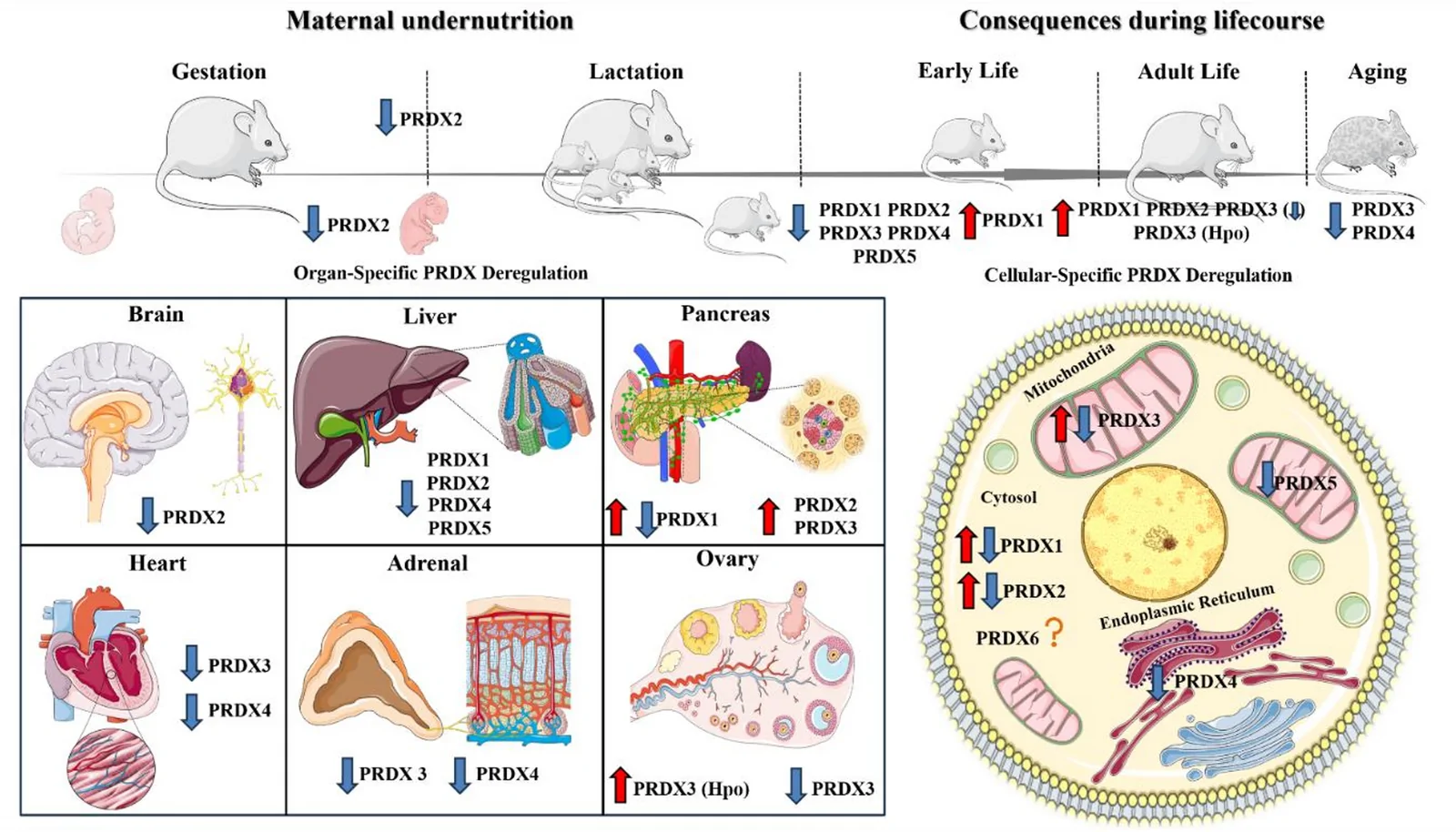
Schematic representation of organ- and cell-specific peroxiredoxin (PRDX) dysregulation induced by maternal malnutrition. Maternal malnutrition during gestation and/or lactation leads to organ-specific alterations in PRDX expression, including the brain, liver, pancreas, heart, adrenal gland, and ovary. At the subcellular level, PRDX dysregulation affects mitochondria, cytosol, and endoplasmic reticulum, indicating a broad disruption of antioxidant homeostasis and redox signaling pathways from early development to aging. The Figure was composed using some illustrations from Servier Medical Art (https://smart.servier.com), licensed under CC BY 4.0 (https://creativecommons.org/licenses/by/4.0/)

Chronic hypothyroidism induced by diet led to a prolonged decrease in plasma thyroid hormone levels, compromising both ovarian follicular reserve and ovulation in prepubertal (12-day-old) and adult (64- and 120-day-old) rats. In adult animals, the number of preantral and antral follicles was reduced, while the proportion of atretic follicles increased, resulting in a diminished ovulation rate. At 120 days of age, hypothyroid rats exhibited significantly higher mRNA and protein levels of SOD1, whereas CAT levels, as well as the expression of *Prdx3*, thioredoxin reductase 1 (*Txnrd1*), and uncoupling protein 2 (*Ucp2*), were decreased. A downward trend was also observed for glutathione peroxidase 3 (*Gpx3*) and glutathione S-transferase mu 2 (*Gstm2*). Moreover, increased immunostaining for the oxidative stress marker 4-hydroxynonenal was detected. Together, these results indicate that chronic hypothyroidism, when initiated during the fetal or neonatal period, reduces ovulation and impairs the ovarian antioxidant defense system [40]. The general findings about maternal undernutrition and PRDXs were compiled in Table 1; Fig. 3.

**Table 1:** Maternal undernutrition and PRDXs effects on the offspring

Main results	Exposition, Macromolecules and, Percentage	Period	Model and organ	Authors
Decreased PRDX1 - Male rats, Decreased PRDX4 - Females rats. Early Life.	Low Protein Diet (6% protein).	Gestation and Lactation.	*Sprague Dawley* rats - Liver.	[17]
Decreased PRDX3 early in life. Decreased PRDX3 and PRDX4 in aging. Male rats.			*Sprague Dawley* rats - Adrenal Gland.	[20]
Decreased PRDX4.			Sprague Dawley rats - Heart.	[14]
Maternal decreased hepatic *Prdx2*. Decreased *Prdx2* early in life.	Low Protein Diet (60% protein of control group: ME = 9.28 MJ/kg and CP = 7.5%) and Energy-Restricted Diet (60% energy of control group: ME = 5.75 MJ/kg and CP = 12.6%).	Gestational day 90 to 145.	Liuyang Blacks goats - Plasma and immune tissues (liver, thymus, spleen, and jejunum).	[33]
Decreased PRDX2 and increased after taurine treatment. Early life.	Low Protein Diet (8% protein, 79% carbohydrate and 13% fat) and taurine supplement (added starting from the 12th day of pregnancy).	Gestation.	*Sprague Dawley* rats – Brain.	[34]
Increased hyperoxidized *Prdx3*. Decreased *Prdx3*. Adult life.	Teklad Global 18% Protein Diet − 50% of the control intake.	Gestation, and/or lactation.	Wistar rats – Ovary.	[35]
Decreased PRDX5 activity in the liver. Decreased PRDX1 and increased PRDX2 in the pancreas. Early life.	Isocaloric low protein diet: 8% protein.	Gestation or Gestation and Lactation.	Wistar rats - Pancreas and Liver.	[36]
Increased PRDX1 and PRDX3. Adult life.	Isocaloric low-protein (8% protein) diet.	Gestation.	Wistar rats - Pancreatic islets.	[37]
Decreased PRDX3. CoQ10 supplementation increased PRDX3. Adult life.		Pregnancy to postnatal day 3.	Wistar rats – Heart.	[38]
Decreased PRDX3. Early life.		Gestation.	Wistar rats – Heart.	[39]
Decreased PRDX3.	Iodide-poor diet supplemented with 0.75% sodium perchlorate in the drinking water to deplete endogenous iodide stores.	Before and during gestation, lactation and post-weaning.	Wistar WU (HsdCpbWU) rats - Ovary.	[40]

## Maternal Overnutrition

Understanding the consequences of maternal overnutrition on offspring health is of critical importance, particularly in the context of contemporary post-industrial dietary patterns characterized by excessive caloric and lipid intake. Surplus macronutrients—especially lipids—can disrupt the maternal–fetal–placental interface and alter breast milk composition, thereby predisposing offspring to long-term metabolic disturbances, including obesity and related cardiometabolic disorders [21, 22]. Given the multifaceted biological roles of PRDXs in redox regulation, mitochondrial function, and inflammatory signaling, their modulation in the setting of maternal overnutrition warrants careful investigation. Here, we summarize the limited available evidence, outlining the potential redox and metabolic alterations described to date while emphasizing the substantial gap in the literature regarding PRDXs as mediators or biomarkers of overnutrition-induced developmental programming.

Experimental data with rats exposed to maternal high fat diet before and during pregnancy and lactation demonstrate that female offspring present changes in intestinal permeability and structure (claudin and Zo-1), impacts on inflammasome mediators (TLR4, TGFb, IL1b, IL18, and IL6), and increased serum TNFα levels, in addition to antioxidant unbalance (increased catalase, PRDX4, and superoxide dismutase 1 in the ileum) [41]. In an interesting model associating the uncommonly exacerbated diet, maternal gliadin peptide intake by *Caenorhabditis elegans* causes decreased oxidative stress resistance. Surviving offspring developed from these oocytes exhibited decreased oxidative stress resistance, besides promoting DAF-16 translocation and the expression of its downstream targets, such as PRDX3 and gst-4 [42].

**Fig. 4:**
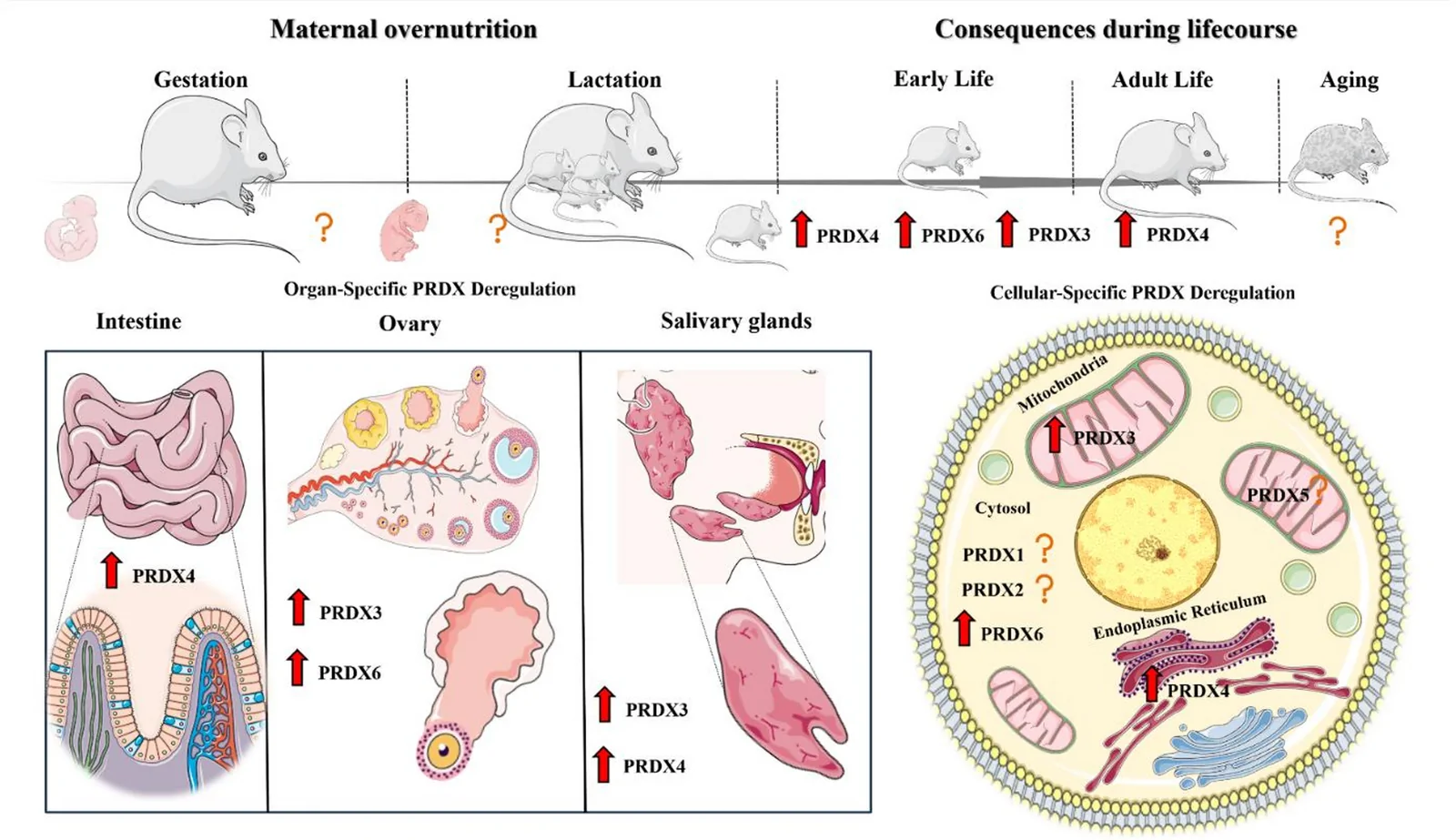
Schematic representation of the organ- and cell-specific dysregulation of peroxiredoxins (PRDX) induced by maternal overnutrition. Maternal malnutrition during gestation and/or lactation leads to organ-specific changes in PRDX expression, including in the intestines, ovary, and submandibular glands. At the subcellular level, PRDX dysregulation affects mitochondria, cytosol, and endoplasmic reticulum, indicating a broad disruption of antioxidant homeostasis and redox signaling pathways from early development to aging. The Figure was composed using some illustrations from Servier Medical Art (https://smart.servier.com), licensed under CC BY 4.0 (https://creativecommons.org/licenses/by/4.0/)

Evaluating the effects of a Western diet (rich in fat/sugar), Morzel et al. (2018) observed, from the proteomic profile of the submandibular gland of male and female pups in early life, impacts on proteins involved in protein quality control (e.g., PDIA3, P4HB, HSPA5), microtubule proteins (TUBA1C, TUBB4B, TUBA4A, ACTB, and ACTG2), as well as oxidative stress proteins (GPX1, GSTM2, GST2, PRDX3 and PRDX4), and an increase in the molecular function of antioxidant activity [43]. The general findings about maternal overnutrition and PRDXs were compiled in Table 2; Fig. 4.

**Table 2:** Maternal overnutrition and PRDXs effects on the offspring

Main results	Exposition, Macromolecules, and percentage	Period	Model and organ	Authors
Increased PRDX4. Female adult mice.	High-fat diet (26.2% protein, 26.3% carbohydrate and 34.9% fat with 60% of energy from fat).	8 weeks before and during pregnancy and lactation.	NOD/ShiLtJ mice – Intestine.	[41]
Increased PRDX3 and PRDX6. Early life.	0 or 3 µM GP at the L4 stage for 3 days. The day-3 adults’ laid eggs for 24 h, and the hatched L1-staged F1 progeny were incubated in M9 buffer containing 100 mM paraquat (1,1’-dimethyl-4,4’-bipyridinium dichloride, Sigma-Aldrich, St. Louis, MO, USA) for 6 h at 20 °C.	Gestation.	*Caenorhabditis elegans* - Gonads	[42]
Male and female offspring rats at weaning - Increased PRDX3 and PRDX4.	High-fat/high-sugar diet (“Western diet” with 21% beef fat and 30% sucrose).	Gestation and lactation.	Sprague Dawley rats – Submandibular glands.	[43]

## Discussion and Insights

This study provides several mechanisms affected by maternal malnutrition, in undernutrition or overnutrition conditions, and their effects on the descendants, specifically from different tissues and PRDX isoforms. Still, it is notable that few studies have investigated the correlation between maternal malnutrition and effects on the PRDXs; however, the DOHaD and the crucial potential role of PRDXs on the healthy or disease are two growing contexts, and here we pretend to demonstrate that we know about the presented studies and future perspectives and possibilities to translational investigations, to understand if PRDXs are associated to metabolic-fetal-programming in maternal diet adversities conditions.

Specifically, when considering PRDXs as candidate biomarkers within the DOHaD framework, they can be conceptualized as redox-regulatory molecules that modulate cellular and biochemical homeostasis in response to early-life environmental challenges. Beyond serving as indicators of oxidative imbalance, PRDXs participate directly in signaling pathways that regulate proliferation, apoptosis, mitochondrial function, and inflammatory responses—central processes to developmental programming. Adverse nutritional exposures during critical windows of development may epigenetically reprogram PRDX expression, thereby establishing persistent alterations that extend from early life into adulthood and potentially throughout the lifespan. In this context, PRDXs meet key criteria for DOHaD biomarkers. First, they demonstrate exposure sensitivity, as their expression is modulated by the severity, duration, and timing of malnutrition or overnutrition (e.g., during gestation and/or lactation). Second, they exhibit biological plausibility, given their integral role in redox signaling and metabolic programming. Third, they hold predictive and functional relevance, since sustained dysregulation of PRDX expression may compromise cellular homeostasis, exacerbate oxidative stress–inflammatory crosstalk, and predispose tissues to metabolic dysfunction or chronic disease later in life. Thus, within a programming and epigenetic framework, PRDXs represent not merely markers of metabolic programming but mechanistically relevant mediators that bridge early nutritional insults with long-term alterations in cellular function and disease susceptibility. However, it is essential to note that the studies found elucidate experimental, rather than epidemiological, aspects; therefore, candidates for mechanistic mediators with the potential to be evaluated as validated predictive biomarkers in clinical contexts were included here.

### Maternal Undernutrition and PRDXS

Regarding organ-specific biomarkers associated with the potential metabolic-antioxidant programming of maternal malnutrition and an in silico analysis approach, we observed temporal effects of different PRDX isoforms with varying modulations of expression. As a starting point, maternal hepatic *Prdx2* mRNA expression decreased in response to undernutrition [17, 34]. Specifically, regarding offspring, the evaluated studies demonstrate that maternal malnutrition decreased brain PRDX2 during fetal life [36], reduced cardiac PRDX3 and PRDX4 [14, 38, 39], imbalanced hepatic PRDX1, and decreased *Prdx2* mRNA expression in males, while decreasing hepatic PRDX4 in females early in life [17]. In pancreatic islets, there was an increase in PRDX1, PRDX2, and PRDX3 [36, 37], while the adrenal gland showed a decrease in PRDX3 (early in life) and a decrease in PRDX3 and PRDX4 in males during aging [20]. In the ovary, there was an increase in hyperoxidized *Prdx3* and a reduction in ovarian *Prdx3* mRNA levels [35, 40]. Maternal malnutrition induced organ- and organelle-specific PRDX alterations, revealing a distinct and sex-specific redox programming. The imbalance of hepatic PRDX1 (cytosolic), decreased cytosolic PRDX2 (brain and liver), mitochondrial PRDX3 (heart, adrenal, ovary, and pancreas) and PRDX5 (liver), and endoplasmic reticulum PRDX4 (liver, heart, and adrenal gland) suggest impaired antioxidant defenses in key metabolic tissues, while the increase in PRDX1–3 in pancreatic islets indicates compensatory redox activation that may be an effect of insulinemia impairment. These studies not only provide an important overview of the effects of maternal malnutrition on PRDX, but also possible other mechanisms affected in specific cellular regions, highlighting the potential for future studies investigating signaling pathways and broader aspects that enable translational and epidemiological studies in this regard. Furthermore, evaluation of other PRDX isoforms under such dietary challenge (PRDX6), as well as the effects on other organs (e.g., testes, thyroid, hypothalamus, pituitary, muscle), is necessary to truly understand whether PRDX are organ-wide hallmarks and biomarkers of maternal malnutrition and the potential developmental origins of the disease.

### Maternal Overnutrition and PRDXS

Within the context observed in our review and in silico analysis, we found few studies that effectively evaluate the effects of maternal overnutrition on PRDXs. Nevertheless, from the few studies available, we can highlight the impacts on the gonads [42], exocrine glands (salivary glands) [43], and small intestine [41] regarding mitochondrial PRDX3, endoplasmic reticulum PRDX4, and cytosolic and membrane-bound PRDX6. Clearly, other organs and other PRDXs should be investigated in the context of maternal overnutrition and its effects on the offspring, but the few existing studies already show potential impacts on energy metabolism, protein synthesis, and vesicular transport, as well as cytosolic enzymatic activity. Here, we can only extrapolate a few future perspectives, perhaps some in line with studies on maternal undernutrition, but there is certainly a direct effect on mitochondria and the endoplasmic reticulum in both contexts, identifying these organelles as potential biomarker organelles for the adverse effects of maternal malnutrition on different organs and systems in the offspring.

### Future Ivestigation and Perspectives

Given the studies observed, it is clear that the PRDX5 and 6 isoforms are underexplored within the thematic scope of this review. Specifically, future studies should synergistically investigate the organ-specific impacts of maternal undernutrition or overnutrition on offspring, encompassing other organs, possible omics mechanisms (epigenome, transcriptome, proteome, metabolome), and even single-cell aspects based on evidence-based technologies and the search for biomarkers - highlighting the potential role of PRDXs in the developmental origins of health or disease. To establish the synergistic characteristic, these future studies should focus efforts on understanding other altered cytosolic mechanisms associated with PRDX1, PRDX2, and PRDX6, such as redox signaling control (e.g., glutathiones, pentose phosphate pathway, NADPH, non-classical antioxidant enzymes) and lipid bilayer homeostasis (lipid peroxidation, transporters, receptors, insertion of glycoproteins and glycolipids into the non-cytosolic face of the membrane), in addition to extrapolating the effects possibly associated with the action of enzymes necessary for anabolic or catabolic aspects.

### Mitochondrial Point of View

PRDX3 and PRDX5 stand out, requiring the investigation of other mechanisms of energy deregulation (e.g., pyruvate dehydrogenase, coenzymes, citrate synthase, and other allosteric or non-allosteric enzymatic actions, formation of electron transporters - FADH, NADH2, b-oxidation enzymes, which act on ketogenic and glucogenic amino acids, integrity of complexes I, II, III and IV of the electron transport chain, among others) and aspects of programmed cell death via apoptosis (e.g., activation of caspases 3, 9, BAX, inactivation of BCL2) and even autophagic elements (e.g., LC3, Beclin, p63).

### Cellular Point of View

Cellular components and biomolecules associated with the endoplasmic reticulum should also be investigated, based on their interaction with PRDX4, such as protein folding, hormone secretion, and exocytosis. Specific translocators (signal recognition particles, scramblases, glycosylators, sulfators, among others) could be investigated, as well as the role of epigenetic components in protein synthesis mechanisms or even the degradation of misfolded proteins (e.g., ubiquinone proteasome). Finally, a thorough examination of the peroxisomal role of PRDX5 and its ability to detoxify organic peroxides is needed, allowing for the investigation of a wide range of peroxisomal enzymes.

### Epigenetic Point of View

In light of the DOHaD concept, the epigenetic effects on PRDXs (e.g., miRNAs, long noncoding RNAs, nuclear RNAs, Piwi RNAs, among others) should be investigated, as well as how this can modulate PRDX-isoform-specific gene expression (e.g., DNMT, DNA methylation, CPG islands, acetylation, histones), splicing, transcription, translation, and function of these molecules. Obviously, the biological complexity is unimaginable; however, we propose a few key mechanisms potentially associated with the different roles of the different PRDX isoforms. If based on omics profiling, this will certainly provide a broad yet personalized understanding of the true role of PRDXs on different dysregulated molecules in maternal malnutrition, as well as a true understanding of whether PRDXs truly are DOHaD biomarkers, from the perspective of potentiating or mitigating adverse consequences.

### Are PRDXS Biomarkers on the DOHaD Concept and Maternal Malnutrition?

Some studies elucidate the specific isoforms of PRDXs in certain pathological conditions, such as the epidemiological correlation between serum PRDX4 and chronic diseases [44] and nephropathy in patients with type 2 diabetes [45], while PRDX2 has been associated as a biomarker of poor neurological prognosis and delayed cerebral ischemia [46]. In experimental investigations, PRDX1 was associated with the aggravation of renal injury [47] and colon cancer [48]. PRDX6 was a potential biomarker of lung adenocarcinoma [49] and as a biomarker of brain injury [50]. Furthermore, PRDX3 was correlated with hepatocellular carcinoma [51], while PRDX1 was proposed as a biomarker for abdominal aortic aneurysm [52]. This overview is quite interesting, as it elevates PRDXs as key molecules in the context of health or disease, and highlights their recently established vital role, emphasizing the need for mechanistic studies that thoroughly investigate the role of these biological molecules in different organ- and cell-specific cellular, molecular, biochemical, and metabolic pathways. However, considering the small number of studies on the DOHaD approach regarding maternal diet and PRDXs in offspring, we cannot yet propose that PRDXs are biomarkers of metabolic programming. On the other hand, the studies presented are interesting and provide fertile ground for future research in this area, enabling the establishment of PRDXs and associated epigenetic factors as DOHaD biomarkers.

### Translational Challenges

Considering the evidence discussed, a major gap in the field of maternal malnutrition and PRDXs within the DOHaD framework is the scarcity of clinical investigations that explicitly validate these molecules as biomarkers of metabolic programming. While experimental models consistently demonstrate that PRDXs are sensitive to early nutritional insults and mechanistically linked to redox and metabolic pathways, their translational consolidation in human cohorts remains limited.

In this context, the present review offers a structured foundation to guide future translational and epidemiological research. The experimental studies summarized herein provide robust mechanistic support for the role of PRDXs in developmental programming, thereby establishing a conceptual framework for clinical validation. Moving forward, well-designed longitudinal and population-based studies will be essential to determine whether the biomarker potential of PRDXs observed in experimental settings can be replicated and consolidated from an epidemiological perspective - analyzing, for example, the PRDXs isoforms expression in the cord blood, placenta, serum human samples-, ultimately strengthening their applicability within the DOHaD paradigm.

### Study Limitaions

The study’s limitations include its focus on PRDXs alone, without deeply analyzing the effects of maternal malnutrition on other cellular, tissue-specific molecular, and metabolic mechanisms. This would likely demonstrate a combined effect of PRDX alterations rather than an isolated one. Another limitation was the exclusive use of PubMed for manuscript searches, but this condition was strategically proposed to find a robust curation of biomedical, experimental, and translational research, which is consistent with the scope of this review. Furthermore, it is important to consider as a limitation the methodological restrictions inherent in the review process, specifically because the search strategy was restricted to selected databases and predefined keywords. Although the terms were carefully chosen to reflect the central components of maternal malnutrition, PRDXs and DOHaD, variations in terminology between publications may have resulted in the omission of eligible articles.

Nevertheless, the objective was to highlight what we know about these molecules from the perspective of DOHaD and dietary adversity, given the growing evidence in both areas. This certainly provides a foundation for future studies in this context. Another obvious limitation was the focus solely on experimental studies, due to the lack of studies evaluating the synergy between maternal malnutrition and PRDX-specific effects on offspring, highlighting a gap in the literature and epidemiological investigations. Finally, a final limitation was not characterizing the effects of PRDXs in different windows of susceptibility beyond negative maternal exposure during pregnancy and lactation. However, we proposed a series of possible mechanisms and even correlations of PRDXs with certain diseases in our discussion, providing a basis for mechanistic, experimental, and translational studies that understand the possible role of PRDXs in health or disease in the face of fetal-metabolic programming in critical windows of development.

## Conclusion

Therefore, this critical-interpretative scoping review underscores the potential of maternal malnutrition - whether overnutrition or undernutrition - to modulate several PRDX isoforms across different cells and organs in a temporal manner throughout offspring development. Based on the experimental evidence, such dysregulations likely affect multiple biological mechanisms, including antioxidant defense, inflammation, cellular regulation, and energy metabolism. The reviewed studies collectively indicate that maternal malnutrition disrupts mitochondrial, cytoplasmic, and endoplasmic reticulum homeostasis, positioning PRDXs as central regulators of redox balance and cellular integrity within this context. These findings reinforce the notion that PRDX isoforms may act not only as markers of oxidative imbalance but also as potential mechanistic mediators of developmental programming. Importantly, this synthesis also reveals a substantial gap in the literature, particularly regarding the translational validation and long-term programming role of PRDXs within the DOHaD framework, underscoring the need for further mechanistic and clinical investigations. Nonetheless, it provides a foundation for future research aimed at determining whether PRDXs may serve as biomarkers of the developmental origins of health and disease, integrating evidence from experimental, translational, and clinical studies. While PRDXs emerge as promising cell- and organ-specific indicators of maternal nutritional stress, their definitive status as biomarkers remains to be established, paving the way for further investigation.

## Declaration of Generative AI and AI-Assisted Technologies in the Writing Process

All the text of the manuscript was initially written without Generative AI. Generative AI was partially used to correct some English formulations or to improve the formulation of a few sentences.

## Supplementary Information

Below is the link to the electronic supplementary material.

Supplementary Material 1

## Data Availability

This article is a review and does not involve the generation or analysis of new datasets. Therefore, data-sharing requirements are not applicable.

## References

[1] Barker, D. J. P., Osmond, C., Golding, J., Kuh, D., & Wadsworth, M. E. J. (1989). Growth in utero, blood pressure in childhood and adult life, and mortality from cardiovascular disease. *British Medical Journal*, *298*(6673), 564–567. 10.1136/BMJ.298.6673.564 2495113 PMC1835925

[2] Barke, T. L., Money, K. M., Du, L., Serezani, A., Gannon, M., Mirnics, K., & Aronoff, D. M. (2019). Sex modifies placental gene expression in response to metabolic and inflammatory stress. *Placenta*, *78*, 1–9. 10.1016/j.placenta.2019.02.008 30955704 PMC6461364

[3] Tamayev, L., Schreiber, L., Marciano, A., Bar, J., & Kovo, M. (2020). Are there gender-specific differences in pregnancy outcome and placental abnormalities of pregnancies complicated with small for gestational age? *Archives of gynecology and obstetrics*, *301*(5), 1147–1151. 10.1007/s00404-020-05514-5 32239281

[4] WHO (2024, March 1). *Malnutrition* [Fact sheet]. World Health Organization. https://www.who.int/news-room/fact-sheets/detail/malnutrition

[5] UNICEF. (n.d.). *Maternal nutrition*. UNICEF. Retrieved September 14 (2025). from https://www.unicef.org/nutrition/maternal

[6] Roseboom, T. J., Painter, R. C., van Abeelen, A. F., Veenendaal, M. V., & de Rooij, S. R. (2011). Hungry in the womb: what are the consequences? Lessons from the Dutch famine. *Maturitas*, *70*(2), 141–145. 10.1016/j.maturitas.2011.06.017 21802226

[7] Nurk, E., Refsum, H., Bjelland, I., Drevon, C. A., Tell, G. S., Ueland, P. M., Vollset, S. E., Engedal, K., Nygaard, H. A., & Smith, D. A. (2013). Plasma free choline, betaine and cognitive performance: the Hordaland Health Study. *The British journal of nutrition*, *109*(3), 511–519. 10.1017/S0007114512001249 22717142

[8] Entringer, S., Buss, C., & Wadhwa, P. D. (2015). Prenatal stress, development, health and disease risk: A psychobiological perspective-2015 Curt Richter Award Paper. *Psychoneuroendocrinology*, *62*, 366–375. 10.1016/j.psyneuen.2015.08.019 26372770 PMC4674548

[9] Miyake, K., Mochizuki, K., Kushima, M., Shinohara, R., Horiuchi, S., Otawa, S., Akiyama, Y., Ooka, T., Kojima, R., Yokomichi, H., Yamagata, Z., & Japan Environment and Children’s Study Group. (2023). Maternal protein intake in early pregnancy and child development at age 3 years. *Pediatric research*, *94*(1), 392–399. 10.1038/s41390-022-02435-8 36624288

[10] Santos, S. A. A., Camargo, A. C., Constantino, F. B., Colombelli, K. T., Mani, F., Rinaldi, J. C., Franco, S., Portela, L. M. F., Duran, B. O. S., Scarano, W. R., Hinton, B. T., Felisbino, S. L., & Justulin, L. A. (2019). Maternal Low-Protein Diet Impairs Prostate Growth in Young Rat Offspring and Induces Prostate Carcinogenesis With Aging. *The journals of gerontology Series A Biological sciences and medical sciences*, *74*(6), 751–759. 10.1093/gerona/gly118 29762647

[11] Mesquita, F. F., Gontijo, J. A., & Boer, P. A. (2010). Expression of renin-angiotensin system signalling compounds in maternal protein-restricted rats: effect on renal sodium excretion and blood pressure. *Nephrology dialysis transplantation: official publication of the European Dialysis and Transplant Association - European Renal Association*, *25*(2), 380–388. 10.1093/ndt/gfp505 19793932

[12] Sene, L. B., Rizzi, V. H. G., Gontijo, J. A. R., & Boer, P. A. (2018). Gestational low-protein intake enhances whole-kidney miR-192 and miR-200 family expression and epithelial-to-mesenchymal transition in rat adult male offspring. *The Journal of experimental biology*, *221*(Pt 10), jeb171694. 10.1242/jeb.171694 29789348

[13] Naia Fioretto, M., Maciel, F. A., Barata, L. A., Ribeiro, I. T., Basso, C. B. P., Ferreira, M. R., Dos Santos, S. A. A., Mattos, R., Baptista, H. S., Portela, L. M. F., Padilha, P. M., Felisbino, S. L., Scarano, W. R., Zambrano, E., & Justulin, L. A. (2024). Impact of maternal protein restriction on the proteomic landscape of male rat lungs across the lifespan. *Molecular and cellular endocrinology*, *592*, 112348. 10.1016/j.mce.2024.112348 39218056

[14] Sobrinho Lemos, L., Naia Fioretto, M., Tenori Ribeiro, I., Annibal Barata, L., Alessandra Maciel, F., Leonardo Fagundes, F., Mattos, R., Portela, F., Barboza, L. M., de Oliveira, J. M. S., Emílio, B., de Almeida, K., Dos Santos, A., Lima, S. A. H., de Arruda Miranda, C. A., Zambrano, J. R., E., & Justulin, L. A. (2025). Maternal Protein Restriction promotes Cardiac Disorders by Disrupting Heart Developmental Morphophysiology in Young Male Offspring Rats. Experimental cell research, 114795. Advance online publication. 10.1016/j.yexcr.2025.114795 41106765

[15] Ribeiro, I. T., Fioretto, M. N., Santos, D., Colombelli, S. A. A., Portela, K. T., Niz Alvarez, L. M. F., de Magalhães Padilha, M. V., Delgado, P., Marques, A. Q., Bosqueiro, M. V. L. S. G., Seiva, J. R., Barbisan, F. R. F., de Andrade Paes, L. F., Zambrano, A. M., E., & Justulin, L. A., Jr (2024). Maternal protein restriction combined with postnatal sugar consumption alters liver proteomic profile and metabolic pathways in adult male offspring rats. *Molecular and cellular endocrinology*, *592*, 112316. 10.1016/j.mce.2024.112316 38880278

[16] Ribeiro, I. T., Fioretto, M. N., Santos, D., Alvarez, S. A. A., Portela, M. V. N., Mattos, L. M. F., Sebastian, R., Vitali, H. B., Seiva, P. M., Barbisan, F. R. F., Lima, L. F., Damasceno, C. A. H., Zambrano, D. C., E., & Justulin, L. A. (2025). Maternal protein restriction and postnatal sugar consumption increases inflammatory response and deregulates metabolic pathways in the liver of male offspring rats with aging. *Molecular and cellular endocrinology*, *599*, 112484. 10.1016/j.mce.2025.112484 39900277

[17] Franzolin, A. M. L., Fioretto, M. N., Ribeiro, I. T., Maciel, F. A., Barata, L. A., Vitali, P. M., Magosso, N., Fagundes, F. L., Emílio-Silva, M. T., Lima, H., Scarano, C. A., W. R., & Justulin, L. A. (2025). Maternal protein restriction compromises hepatic phenotype and antioxidant defense in postweaning male rats, while females exhibit resilience. *Biochemical and biophysical research communications*, *766*, 151873. 10.1016/j.bbrc.2025.151873 40300334

[18] Dos Santos, I. B. L., Fioretto, M. N., Jorge, M. S., Barata, L. A., Ribeiro, I. T., Franzolin, A. M. L., Stoppa, E. G., Mattos, R., Portela, L. M. F., Emílio Silva, M. T., Santos, D., de Arruda, S. A. A., Miranda, J. R., Lima, H., C. A., & Justulin, L. A. (2025). Maternal protein restriction impairs intestinal morphophysiology and antioxidant system in young male offspring rats. *Experimental cell research*, *446*(1), 114464. 10.1016/j.yexcr.2025.114464 39986598

[19] Valente, J. S., Colombelli, K. T., Pereira, L. L., Perez, É. S., Zanella, T., Delgado, B. T., Fioretto, A. Q., Padovani, M. N., Vechetti, C. R., Damasceno, I. J., Justulin, D. C., L. A.,&, & Dal-Pai-Silva, M. Jr (2025). Aerobic exercise acts differentially on proteins from glucose and glycogen pathways in the SOL and PL muscles of offspring rats submitted to a low-protein maternal diet. *Biochemical and biophysical research communications*, *752*, 151483. 10.1016/j.bbrc.2025.151483 39954356

[20] Fioretto, M. N., Barata, L. A., de Andrade Felipe, V. A., Dos Santos, S. A. A., Maciel, F. A., Ribeiro, I. T., Mattos, R., Baptista, H. S., Bueno, G., Fagundes, F. L., Portela, L. M. F., Scarano, W. R., Seiva, F. R. F., Lima, C. A. H., & Justulin, L. A. (2025). Long-term effects of maternal protein restriction on adrenal proteomic profile and steroidogenesis in male offspring rats. *Cellular signalling*, *130*, 111707. 10.1016/j.cellsig.2025.111707 40032160

[21] Denizli, M., Capitano, M. L., & Kua, K. L. (2022). Maternal obesity and the impact of associated early-life inflammation on long-term health of offspring. *Frontiers in cellular and infection microbiology*, *12*, 940937. 10.3389/fcimb.2022.940937 36189369 PMC9523142

[22] Eitmann, S., Mátrai, P., Németh, D., Hegyi, P., Lukács, A., Bérczi, B., Czumbel, L. M., Kiss, I., Gyöngyi, Z., Varga, G., Balaskó, M., & Pétervári, E. (2022). Maternal overnutrition elevates offspring’s blood pressure-A systematic review and meta-analysis. *Paediatric and perinatal epidemiology*, *36*(2), 276–287. 10.1111/ppe.12859 35041216 PMC9305555

[23] Eitmann, S., Németh, D., Hegyi, P., Szakács, Z., Garami, A., Balaskó, M., Solymár, M., Erőss, B., Kovács, E., & Pétervári, E. (2020). Maternal overnutrition impairs offspring’s insulin sensitivity: A systematic review and meta-analysis. *Maternal & child nutrition*, *16*(4), e13031. 10.1111/mcn.13031 32567808 PMC7503101

[24] Wang, Y., Wang, K., Du, M., Khandpur, N., Rossato, S. L., Lo, C. H., VanEvery, H., Kim, D. Y., Zhang, F. F., Chavarro, J. E., Sun, Q., Huttenhower, C., Song, M., Nguyen, L. H., & Chan, A. T. (2022). Maternal consumption of ultra-processed foods and subsequent risk of offspring overweight or obesity: results from three prospective cohort studies. *BMJ (Clinical research ed)*, *379*, e071767. 10.1136/bmj-2022-071767 36198411 PMC9533299

[25] Salazar, A. M., Sordo, M., Navarrete-Monroy, E., Pánico, P., Díaz-Villaseñor, A., Montúfar-Chaveznava, R., Caldelas, I., & Ostrosky-Wegman, P. (2021). Maternal overnutrition before and during pregnancy induces DNA damage in male offspring: A rabbit model. *Mutation research Genetic toxicology and environmental mutagenesis*, *865*, 503324. 10.1016/j.mrgentox.2021.503324 33865538

[26] Meneghini, M. A., Galarza, R. A., Quiroga, F., J. P., & Faletti, A. G. (2022). Diet-induced maternal obesity and overnutrition cause a decrease in the sperm quality of the offspring. *The Journal of nutritional biochemistry*, *103*, 108966. 10.1016/j.jnutbio.2022.108966 35181443

[27] Gawlińska, K., Gawliński, D., Filip, M., & Przegaliński, E. (2021). Relationship of maternal high-fat diet during pregnancy and lactation to offspring health. *Nutrition reviews*, *79*(6), 709–725. 10.1093/nutrit/nuaa020 32447401

[28] Zhang, L., Zou, W., Zhang, S., Wu, H., Gao, Y., Zhang, J., & Zheng, J. (2024). Maternal high-fat diet orchestrates offspring hepatic cholesterol metabolism via MEF2A hypermethylation-mediated CYP7A1 suppression. *Cellular & molecular biology letters*, *29*(1), 154. 10.1186/s11658-024-00673-8 39695937 PMC11654142

[29] Tricco, A. C., Lillie, E., Zarin, W., O’Brien, K. K., Colquhoun, H., Levac, D., Moher,D., Peters, M. D. J., Horsley, T., Weeks, L., Hempel, S., Akl, E. A., Chang, C., McGowan,J., Stewart, L., Hartling, L., Aldcroft, A., Wilson, M. G., Garritty, C., Lewin, S.,… Straus, S. E. (2018). PRISMA Extension for Scoping Reviews (PRISMA-ScR): Checklist and Explanation. Annals of internal medicine, 169(7), 467–473. 10.7326/M18-0850. 30178033

[30] Chen, E. Y., Tan, C. M., Kou, Y., Duan, Q., Wang, Z., Meirelles, G. V., & Clark, N. R. (2013). & Ma’ayan, A. Enrichr: interactive and collaborative HTML5 gene list enrichment analysis tool. BMC bioinformatics, 14, 128. 10.1186/1471-2105-14-128 PMC3637064 23586463

[31] Kuleshov, M. V., Jones, M. R., Rouillard, A. D., Fernandez, N. F., Duan, Q., Wang, Z., Koplev, S., Jenkins, S. L., Jagodnik, K. M., Lachmann, A., McDermott, M. G., Monteiro, C. D., Gundersen, G. W., & Ma (2016). Enrichr: a comprehensive gene set enrichment analysis web server 2016 update. *Nucleic acids research*, *44*(W1), W90–W97. 10.1093/nar/gkw377. ‘ayan. 27141961 PMC4987924

[32] Xie, Z., Bailey, A., Kuleshov, M. V., Clarke, D. J. B., Evangelista, J. E., Jenkins, S. L., Lachmann, A., Wojciechowicz, M. L., Kropiwnicki, E., Jagodnik, K. M., & Jeon, M. (2021). & Ma’ayan, A. Gene Set Knowledge Discovery with Enrichr. Current protocols, 1(3), e90. 10.1002/cpz1.90 PMC8152575 33780170

[33] He, Z. X., Sun, Z. H., Tan, Z. L., Tang, S. X., Zhou, C. S., Han, X. F., Wang, M., Wu, D. Q., Kang, J. H., & Beauchemin, K. A. (2012). Effects of maternal protein or energy restriction during late gestation on antioxidant status of plasma and immune tissues in postnatal goats. *Journal of animal science*, *90*(12), 4319–4326. 10.2527/jas.2012-5088 22952363

[34] Liu, H., Wang, Y., Liu, J., & Fu, W. (2019). Proteomics analysis of fetal growth restriction and taurine–treated fetal growth restriction rat brain tissue by 2D DIGE and MALDI–TOF/TOF MS analysis. *International journal of molecular medicine*, *44*(1), 207–217. 10.3892/ijmm.2019.4182 31115483 PMC6559329

[35] Bernal, A. B., Vickers, M. H., Hampton, M. B., Poynton, R. A., & Sloboda, D. M. (2010). Maternal undernutrition significantly impacts ovarian follicle number and increases ovarian oxidative stress in adult rat offspring. *PloS one*, *5*(12), e15558. 10.1371/journal.pone.0015558 21179452 PMC3001490

[36] Theys, N., Clippe, A., Bouckenooghe, T., Reusens, B., & Remacle, C. (2009). Early low protein diet aggravates unbalance between antioxidant enzymes leading to islet dysfunction. *PloS one*, *4*(7), e6110. 10.1371/journal.pone.0006110 19568427 PMC2699474

[37] Tarry-Adkins, J. L., Chen, J. H., Smith, N. S., Jones, R. H., Cherif, H., & Ozanne, S. E. (2009). Poor maternal nutrition followed by accelerated postnatal growth leads to telomere shortening and increased markers of cell senescence in rat islets. *FASEB journal: official publication of the Federation of American Societies for Experimental Biology*, *23*(5), 1521–1528. 10.1096/fj.08-122796 19126595

[38] Tarry-Adkins, J. L., Blackmore, H. L., Martin-Gronert, M. S., Fernandez-Twinn, D. S., McConnell, J. M., Hargreaves, I. P., Giussani, D. A., & Ozanne, S. E. (2013). Coenzyme Q10 prevents accelerated cardiac aging in a rat model of poor maternal nutrition and accelerated postnatal growth. *Molecular metabolism*, *2*(4), 480–490. 10.1016/j.molmet.2013.09.004 24327963 PMC3854989

[39] Tarry-Adkins, J. L., Martin-Gronert, M. S., Fernandez-Twinn, D. S., Hargreaves, I., Alfaradhi, M. Z., Land, J. M., Aiken, C. E., & Ozanne, S. E. (2013). Poor maternal nutrition followed by accelerated postnatal growth leads to alterations in DNA damage and repair, oxidative and nitrosative stress, and oxidative defense capacity in rat heart. *FASEB journal: official publication of the Federation of American Societies for Experimental Biology*, *27*(1), 379–390. 10.1096/fj.12-218685 23024373

[40] Meng, L., Rijntjes, E., Swarts, H., Bunschoten, A., van der Stelt, I., Keijer, J., & Teerds, K. (2016). Dietary-Induced Chronic Hypothyroidism Negatively Affects Rat Follicular Development and Ovulation Rate and Is Associated with Oxidative Stress. *Biology of reproduction*, *94*(4), 90. 10.1095/biolreprod.115.136515 26962119

[41] Xue, Y., Wang, H., Du, M., & Zhu, M. J. (2014). Maternal obesity induces gut inflammation and impairs gut epithelial barrier function in nonobese diabetic mice. *The Journal of nutritional biochemistry*, *25*(7), 758–764. 10.1016/j.jnutbio.2014.03.009 24775094 PMC4050341

[42] Lee, J. H., Lee, M., Min, H., Youn, E., & Shim, Y. H. (2022). Maternal Gliadin Intake Reduces Oocyte Quality with Chromosomal Aberrations and Increases Embryonic Lethality through Oxidative Stress in a Caenorhabditis elegans Model. *Nutrients*, *14*(24), 5403. 10.3390/nu14245403 36558561 PMC9787971

[43] Morzel, M., Brignot, H., Ménétrier, F., Lucchi, G., Paillé, V., Parnet, P., Nicklaus, S., & Canivenc-Lavier, M. C. (2018). Protein expression in submandibular glands of young rats is modified by a high-fat/high-sugar maternal diet. *Archives of oral biology*, *96*, 87–95. 10.1016/j.archoralbio.2018.08.021 30205238

[44] Geertsema, S., Geertsema, P., Kieneker, L. M., Abdulle, A. E., la Bastide-van Gemert, S., Bakker, S. J. L., Dullaart, R. P. F., Dijkstra, G., Gansevoort, R. T., Faber, K. N., van Goor, H., & Bourgonje, A. R. (2024). Serum peroxiredoxin-4, a biomarker of oxidative stress, associates with new-onset chronic kidney disease: A population-based cohort study. *Redox biology*, *77*, 103408. 10.1016/j.redox.2024.103408 39490314 PMC11550021

[45] Bourgonje, A. R., van Goor, H., Bakker, S. J. L., Hillebrands, J. L., Bilo, H. J. G., Dullaart, R. P. F., & van Dijk, P. R. (2024). Serum peroxiredoxin-4, a biomarker of oxidative stress, is associated with the development of nephropathy in patients with type 2 diabetes (Zodiac-65). *Free radical biology & medicine*, *212*, 186–190. 10.1016/j.freeradbiomed.2023.12.025 38151214

[46] Fu, Z., Dai, J., Lin, Q., Ye, L., Chen, X., Li, Z., Lu, C., Chen, M., Ba, H., Sun, J., & Cai, J. (2025). Serum peroxiredoxin 2 emerges as a predictive biomarker of poor neurological outcome and delayed cerebral ischemia after aneurysmal subarachnoid hemorrhage: a prospective cohort study. *Neurosurgical review*, *48*(1), 412. 10.1007/s10143-025-03565-3 40347370

[47] Li, S., Zhang, Y., Lu, R., Lv, X., Lei, Q., Tang, D., Dai, Q., Deng, Z., Liao, X., Tu, S., Yang, H., Xie, Y., Meng, J., Yuan, Q., Qin, J., Pu, J., Peng, Z., & Tao, L. (2023). Peroxiredoxin 1 aggravates acute kidney injury by promoting inflammation through Mincle/Syk/NF-κB signaling. *Kidney international*, *104*(2), 305–323. 10.1016/j.kint.2023.04.013 37164261

[48] Jaiswal, S., Joshi, B., Chen, J., Wang, F., Dame, M. K., Spence, J. R., Newsome, G. M., Katz, E. L., Shah, Y. M., Ramakrishnan, S. K., Li, G., Lee, M., Appelman, H. D., Kuick, R., & Wang, T. D. (2022). Membrane Bound Peroxiredoxin-1 Serves as a Biomarker for *In Vivo* Detection of Sessile Serrated Adenomas. *Antioxidants & redox signaling*, *36*(1–3), 39–56. 10.1089/ars.2020.8244 34409853 PMC8792500

[49] Li, B. Z., Bai, H. H., Tan, F. W., Gao, Y. B., & He, J. (2021). Identification of peroxiredoxin 6 as a potential lung-adenocarcinoma biomarker for predicting chemotherapy response by proteomic analysis. *Journal of biological regulators and homeostatic agents*, *35*(2), 537–546. 10.23812/20-683-A 33631924

[50] Buonora, J. E., Mousseau, M., Jacobowitz, D. M., Lazarus, R. C., Yarnell, A. M., Olsen, C. H., Pollard, H. B., Diaz-Arrastia, R., Latour, L., & Mueller, G. P. (2015). Autoimmune Profiling Reveals Peroxiredoxin 6 as a Candidate Traumatic Brain Injury Biomarker. *Journal of neurotrauma*, *32*(22), 1805–1814. 10.1089/neu.2014.3736 25938937 PMC4651056

[51] Qiao, B., Wang, J., Xie, J., Niu, Y., Ye, S., Wan, Q., & Ye, Q. (2012). Detection and identification of peroxiredoxin 3 as a biomarker in hepatocellular carcinoma by a proteomic approach. *International journal of molecular medicine*, *29*(5), 832–840. 10.3892/ijmm.2012.916 22344546

[52] Martinez-Pinna, R., Ramos-Mozo, P., Madrigal-Matute, J., Blanco-Colio, L. M., Lopez, J. A., Calvo, E., Camafeita, E., Lindholt, J. S., Meilhac, O., Delbosc, S., Michel, J. B., de Vega, M., Egido, J., & Martin-Ventura, J. L. (2011). Identification of peroxiredoxin-1 as a novel biomarker of abdominal aortic aneurysm. *Arteriosclerosis thrombosis and vascular biology*, *31*(4), 935–943. 10.1161/ATVBAHA.110.214429 21273562

