# Correction: Peroxiredoxins in the Developmental Origins of Health and Disease: Insights from Maternal Under and Overnutrition – A Critical Interpretative Scoping Review

**DOI:** 10.1007/s12013-026-02086-0

**Published:** 2026-04-27

**Authors:** Matheus Naia Fioretto, Luísa Correia Gomes, Flávia Alessandra Maciel, Ana Luiza Silva de Abreu, Ana Luiza Romano Gabriel, Sérgio Luis Felisbino, Luis Antonio Justulin

**Affiliations:** Department of Structural and Functional Biology, Institute of Biosciences, São Paulo State University, Botucatu, SP Brazil

Correction to: *Cell Biochemistry and Biophysics* **X**:XXXX; https://doi.org/10.1007/s12013-026-02061-9; Article published online XX Month XXXX

In the original version of this article, the first author’s name, ‘Matheus Naia Fioretto,’ was inadvertently duplicated in the author list. The correct author order as follows:

Matheus Naia Fioretto, Luísa Correia Gomes, Flávia Alessandra Maciel, Ana Luiza Silva de Abreu, Ana Luiza Romano Gabriel, Sérgio Luis Felisbino, Luis Antonio Justulin

The original article has been corrected.

